# Population-level detection of early loss of kidney function: 7-year follow-up of a young adult cohort at risk of Mesoamerican nephropathy

**DOI:** 10.1093/ije/dyad151

**Published:** 2023-10-31

**Authors:** Marvin Gonzalez-Quiroz, Brianna Heggeseth, Armando Camacho, Amin Oomatia, Ali M Al-Rashed, Yixuan Zhang, Alexander McCreight, Nicholas Jewell, Aurora Aragon, Dorothea Nitsch, Neil Pearce, Ben Caplin

**Affiliations:** Department of Renal Medicine, University College London, London, UK; Wuqu’ Kawoq Maya Health Alliance, Chimaltenango, Guatemala; Department of Mathematics, Statistics, and Computer Science, Macalester College, St Paul, MN, USA; Research Centre on Health, Work and Environment, National Autonomous University of Nicaragua, León (UNAN-Leon), León, Nicaragua; Department of Renal Medicine, University College London, London, UK; Department of Renal Medicine, University College London, London, UK; Department of Mathematics, Statistics, and Computer Science, Macalester College, St Paul, MN, USA; Department of Mathematics, Statistics, and Computer Science, Macalester College, St Paul, MN, USA; Department of Medical Statistics, London School of Hygiene and Tropical Medicine, London, UK; Wuqu’ Kawoq Maya Health Alliance, Chimaltenango, Guatemala; Department of Non-Communicable Disease Epidemiology, London School of Hygiene and Tropical Medicine, London, UK; Department of Medical Statistics, London School of Hygiene and Tropical Medicine, London, UK; Department of Non-Communicable Disease Epidemiology, London School of Hygiene and Tropical Medicine, London, UK; Department of Renal Medicine, University College London, London, UK

**Keywords:** Chronic kidney disease of undetermined cause, chronic kidney disease of non-traditional cause, hidden Markov modelling

## Abstract

**Background:**

Mesoamerican nephropathy is a leading contributor to premature mortality in Central America. Efforts to identify the cause are hampered by difficulties in distinguishing associations with potential initiating factors from common exposures thought to exacerbate the progression of all forms of established chronic kidney disease (CKD). We explored evidence of disease onset or departure from the healthy estimated glomerular filtration rate distribution [departure from ∼eGFR_(healthy)_] in an at-risk population.

**Methods:**

Two community-based cohorts (adults aged 18–30 years, *n* = 351 and 420) from 11 rural communities in Northwest Nicaragua were followed up over 7 and 3 years respectively. We examined associations with both (i) incident CKD and (ii) the time point of departure from ∼eGFR_(healthy)_, using a hidden Markov model.

**Results:**

CKD occurred in men only (male incidence rate: 0.7%/year). Fifty-three (out of 1878 visits, 2.7%) and 8 (out of 1067 visits, 0.8%) episodes of probable departure from ∼eGFR_(healthy)_ occurred in men and women, respectively. Cumulative time in sugarcane work and symptoms of excess occupational sun exposure were associated with incident CKD. The same exposures were associated with probability of departure from ∼eGFR_(healthy)_ in time-updated analyses along with measured and self-reported weight loss, nausea, vomiting and cramps, as well as non-steroidal anti-inflammatory drug use.

**Conclusions:**

CKD burden in this population is high and risk factors for established disease are occupational. Additionally, a syndrome suggesting an alternative exposure is associated with evidence of disease onset supporting a possible separate unknown initiating factor for which further investigation is needed. Interventions to reduce the impact of occupational risks should be pursued meanwhile.

Key Messages

- Mesoamerican nephropathy is a leading cause of death among young adults in rural Central America yet the cause of disease remains unclear.
- Distinguishing initiating from exacerbating factors is challenging when studying the aetiology of Mesoamerican nephropathy as traditional definitions of disease based on estimates of kidney dysfunction only capture established/advanced cases (i.e. chronic kidney disease defined by an estimated glomerular filtration rate of <60 mL/min/1.7 m^2^).
- Using a novel hidden Markov modelling approach, we are able to identify episodes of sustained kidney function loss from levels in the healthy range occurring commonly in men and less commonly in women using testing data from a community-based population-representative cohort study of young adults conducted in rural Northwest Nicaragua over the last 7 years.
- Alongside occupational risk factors that are also associated with established disease, we observe associations between cramps, nausea and vomiting along with self-reported and measured weight loss and these episodes of early sustained kidney function loss.
- The early sustained kidney function loss is consistent with the initial stages of Mesoamerican nephropathy and the associations with systemic upset and weight loss, independently of occupation, support a possible as yet unidentified initiating cause of disease.

## Introduction

Chronic kidney disease (CKD) of undetermined cause (CKDu) occurs in a number of rural low- and middle- income (LMIC) settings.^1^ CKDu also occurs in Central America, where it is termed Mesoamerican nephropathy (MeN), and it is a leading contributor to premature mortality.^2^

There remains much debate as to the cause of MeN/CKDu. A number of investigators have promoted the idea that physiological heat stress, due to poor work conditions in the agricultural sector, is the primary cause of disease^3^ with studies demonstrating those with greater occupational heat exposure at the highest risk of kidney injury across the work season.^4^^,^^5^ Others have challenged this idea, arguing that heat stress represents an exacerbating factor for CKD progression in general rather than a primary cause of CKDu or MeN.^6^

Given the range of potential kidney disease exacerbating factors that individuals are exposed to in the at-risk areas (e.g. nephrotoxin use or episodes of dehydration), uncovering the aetiology of MeN likely requires observation of early signs of kidney damage. This is challenging, as haematuria and proteinuria, which are useful early markers of many forms of kidney disease, are uncommon in MeN.^7^ Case identification therefore relies on measures of kidney (dys)function, i.e. the estimated glomerular filtration rate (eGFR). Yet, the use of eGFR-based definitions of kidney disease is not straightforward. First, because there is substantial variation in eGFR values between healthy individuals^8^ and, second, because healthy young adults have considerable renal reserve,^9^ substantial underlying kidney damage can already be present with an eGFR that does not meet the internationally accepted threshold for CKD (i.e. <60 mL/min/1.7 m^2^).^10^

Taken together, this suggests that alongside exploring risk factors for established disease, i.e. CKD, approaches aimed at identifying associations with sustained loss of kidney function whilst the eGFR is in the normal or near-normal range, i.e. identifying an individual’s departure from a state of ‘kidney health’, might be useful when attempting to gain aetiological insight. We now report findings from the extended follow-up of our previously described community-based longitudinal study of an initially apparently healthy population who are at risk of MeN,^11^ as well as including results from a more recently recruited cohort. We aimed to examine the occurrence and risk factors for both (i) CKD and (ii) an individual’s departure from a state of ‘kidney health’.

## Methods

Brief methods are described below with details provided in the Supplementary material.

### Study population and study procedures

**Figure 1.**
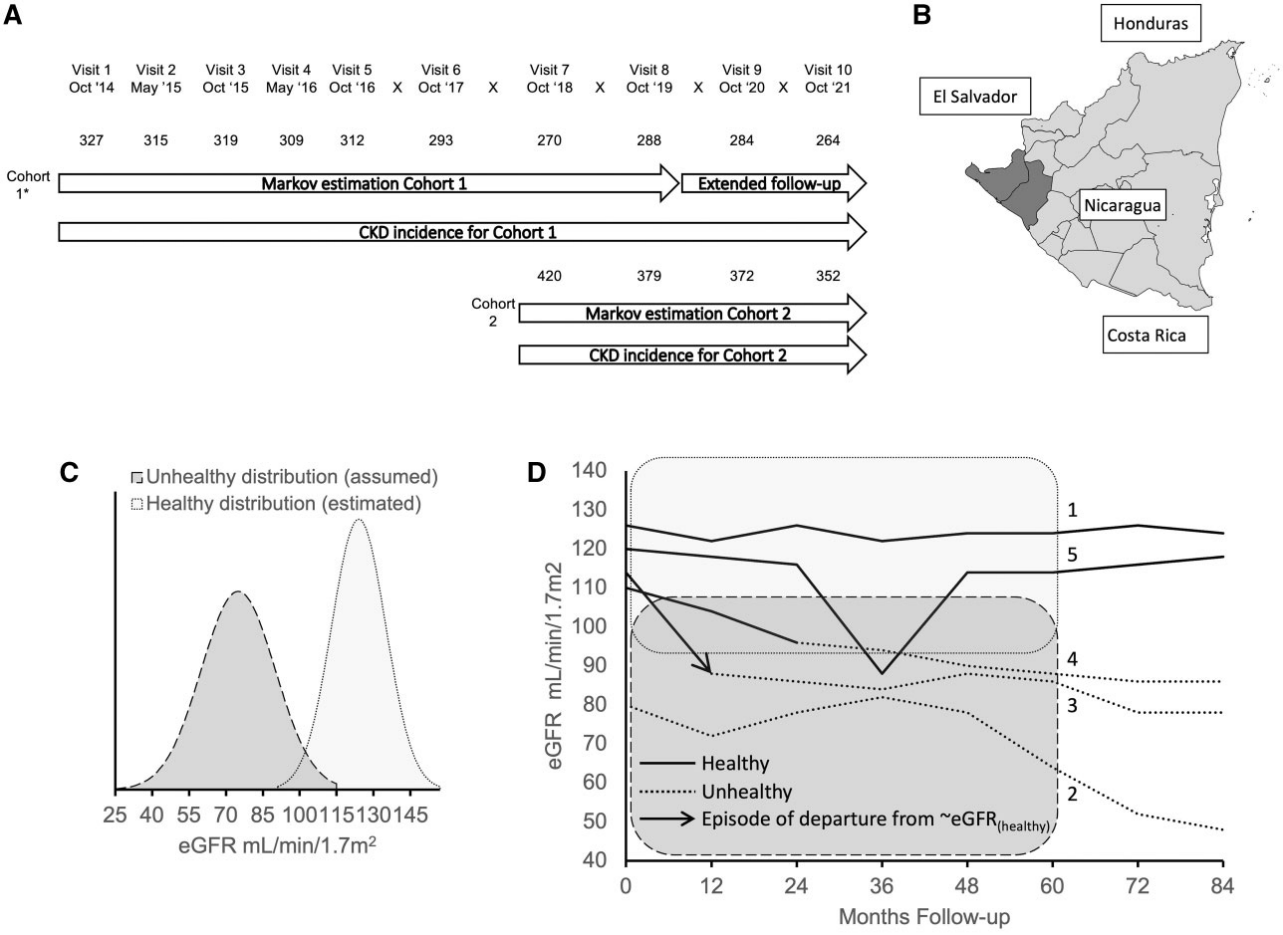
Outline of study design, location and analysis. (A) Recruitment and study visits over the 7-year follow-up with associated analyses described in this report. Numbers show the number of participants with estimated glomerular filtration rate (eGFR) results at each visit. *24 participants from Cohort 1 were recruited at Visit 2. (B) Leon and Chinandega regions highlighted (dark grey) on a map of Nicaragua. (C) Illustration of eGFR distribution of assumed unhealthy (dark grey) and estimated healthy (light grey) kidney states used in the hidden Markov model. The healthy eGFR distribution was estimated empirically. (D) Illustrative eGFR trajectories superimposed on healthy (light grey) and unhealthy (dark grey) Hidden Markov model distributions. Individuals (labelled 1) might be classified as remaining in a healthy kidney state (solid line) throughout. Other individuals (labelled 2) might be classified as in an unhealthy state (dotted line) at all time points. Individuals (labelled 3) who transition between states might be estimated to have a high probability (i.e. >50%) of transitioning from a healthy to unhealthy state since the previous study visit [marked with the arrowhead and referred to as departure from ∼eGFR_(healthy)_ episode in this study]. It is the probability of this departure from ∼eGFR_(healthy)_ at each study visit that is the outcome used in the time-updated risk-factor analyses in this study. Alternatively, an individual may sustain more gradual decline in eGFR (labelled 4) where they are estimated to have lower probabilities of moving from healthy to unhealthy across several time points [i.e. probabilities of >0% and <50% of a departure from ∼eGFR_(healthy)_ event at multiple time points]. Although no single time point of departure from ∼eGFR_(healthy)_ can be identified, these lower probabilities nonetheless contribute to the time-updated risk-factor analyses in this study. Individuals (labelled 5) with an isolated eGFR measure in the unhealthy distribution may remain classified as healthy throughout or revert to a healthy classification after a number of follow-up visits, as the model makes use of all data points in the follow-up period (see text). Again, the departure from ∼eGFR_(healthy)_ probabilities for these individuals contribute to the risk-factor analyses. Individuals estimated to already be in an unhealthy state do not contribute to the risk-factor analyses (dashed lines), i.e. all observations where the posterior probability of an unhealthy state was >50% at the previous study visit were censored. eGFR measures beyond 60 months were not used for the hidden Markov model to provide insight into the consequences of the estimated Markov states on the medium-term eGFR of participants. eGFR, estimated glomerular filtration rate; departure from ∼eGFR_(healthy)_, departure from the healthy eGFR distribution

Participants were invited to take part in a community-based population-representative cohort study in two phases (Cohort 1 and 2) from 11 rural communities in Leon and Chinandega departments, Nicaragua (Figure 1A and B). A detailed rationale and description of the original study design^12^ and the findings of the first 2 years of follow-up^11^ have been published elsewhere.

Inclusion criteria for all cohorts were age 18–30 years and absence of self-reported kidney disease, diabetes or hypertension. Questionnaire data, clinical measurements and biological samples were collected at baseline and then either 6-monthly or annually for a maximum of 7 years. eGFR was calculated using the 2009 CKD-EPI formula.^8^

### CKD-free survival

CKD (Stages 3–5) free survival was estimated using the Kaplan–Meier method with outcome defined as the time of the first of two consecutive eGFR measures of <60 mL/min/1.7 m^2^ (which by virtue of design were ≥6 months apart).

### Hidden Markov modelling

A continuous-time hidden Markov model (HMM) was used based on eGFR measurements over time^13^—an approach well suited for modelling an individual’s movement between a series of states in continuous time, identifying early disease based on longitudinal biomarkers^14–16^ and estimating the time of departure from a healthy kidney state. Two underlying latent kidney states, namely healthy and unhealthy, were assumed, with associated eGFR distributions (Figure 1C and further details in Supplementary material, available as Supplementary data at *IJE* online). The final two visits for Cohort 1 were omitted from the HMM estimates to assess whether there were sustained differences in eGFR after a further 1–2 years of follow-up observed between the group who were classified as remaining healthy and the group classified as transitioning from healthy to unhealthy, as absence of a difference would have suggested that the HMM approach was of limited utility.

Based on the fit HMM, at each study visit, both (i) the posterior probability of being in a healthy or unhealthy state and (ii) the probability of transition from healthy to unhealthy were estimated (Figure 1D). The probability of this transition, termed departure from the healthy eGFR distribution [departure from ∼eGFR_(healthy)_], was defined as the joint probability that an individual is in an unhealthy kidney state at the current visit having transitioned from a healthy state at the previous study visit, i.e. an estimate of the probability that an individual had developed early disease since the last follow-up (Figure 1D).

### Risk-factor analysis

Cohorts were combined for risk-factor analysis. Associations with baseline, cumulative risk or time-updated factors for incident CKD were explored in males only using a Cox proportional-hazards model.

Time-updated risk factors for episodes of departure from ∼eGFR_(healthy)_ were explored using a fractional logit model with the outcome defined as the probability of departure from ∼eGFR_(healthy)_ at each study visit, including all observations in which an individual was estimated to be in a healthy state at the previous visit.

Lastly, a number of exploratory analyses were performed to explore whether the factors associated with the departure from ∼eGFR_(healthy)_ outcome were confounded by nephrotoxic drug use, occupation/occupational environment or water intake using multivariable models. Furthermore, the frequency of these exposures by sex and season was examined.

As this was a hypothesis-generating analysis, no adjustments for multiple testing were made. Analyses were conducted in RStudio, version 2022.02.1 + 461, R Foundation for Statistical Computing, Vienna, Austria and Stata, version 17.0, StataCorp, Texas, USA.

## Results

### Study cohorts

Overall, 351 participants were recruited in Cohort 1 and 420 in Cohort 2. Data on a maximum of 10 and 4 study visits (84 and 36 months of follow-up) were available, respectively, and, overall, 88% of planned study visits were attended (with men overall attending fewer visits). Select cohort characteristics are presented in Table 1 (and a description of cumulative exposure variables over follow-up presented in Supplementary Table S1, available as Supplementary data at *IJE* online).

**Table 1.** Selected baseline demographic and occupational characteristics of study participants stratified by cohort and sex

	Cohort 1		Cohort 2	
Characteristic	Male	Female	Male	Female
*n*	265	86	213	207
Total person-years of follow-up	1640	548	564	583
Community				
1	29 (10.9%)	9 (10.5%)	17 (8.0%)	19 (9.2%)
2	34 (12.8%)	10 (11.6%)	13 (6.1%)	16 (7.7%)
3	27 (10.2%)	11 (12.8%)	24 (11.3%)	19 (9.2%)
4	27 (10.2%)	8 (9.3%)	3 (1.4%)	3 (1.4%)
5	20 (7.5%)	6 (7.0%)	20 (9.4%)	21 (10.1%)
6	42 (15.8%)	12 (14.0%)	18 (8.5%)	19 (9.2%)
7	22 (8.3%)	9 (10.5%)	13 (6.1%)	15 (7.2%)
8	28 (10.6%)	12 (14.0%)	14 (6.6%)	9 (4.3%)
9	36 (13.6%)	9 (10.5%)	44 (20.7%)	38 (18.4%)
10	0	0	29 (13.6%)	26 (12.6%)
11	0	0	18 (8.5%)	22 (10.6%)
Baseline demographics and clinical measures				
Age at baseline (years)	23.3 (3.7)	23.7 (3.5)	22.6 (3.6)	23.5 (3.6)
BMI (kg/m^2^)	23.0 (3.6)	26.5 (6.2)	22.9 (3.3)	26.2 (5.0)
Mean systolic blood pressure	118.7 (9.3)	110.6 (10.8)	121.0 (11.8)	112.9 (10.3)
Mean diastolic blood pressure	69.1 (7.2)	68.4 (7.9)	72.9 (8.6)	71.4 (8.5)
Piped water source only	141 (53.2%)	46 (53.5%)	137 (64.3%)	132 (63.8%)
Family income per month (thousands of Córdobas)	6.0 (4.0–10.0)	5.0 (3.4–8.0)	7.0 (5.0–10.0)	6.0 (4.0–9.2)
Baseline eGFR (mL/min/1.7 m^2^)	122 (112–128)	126 (120–130)	117 (103–126)	123 (114–129)
Follow-up and final eGFR				
Number of study visits completed	9 (7–10)	10 (9–10)	4 (3–4)	4 (4–4)
Participant-level months of follow-up	84 (72–84)	84 (84–84)	36 (36–36)	36 (36–36)
Last eGFR (mL/min/1.7 m^2^)	107 (88–121)	119 (107–126)	112 (96–122)	122 (115–127)
Occupational history at baseline				
Years worked in sugarcane	2 (0–4)	0 (0–0)	0 (0–2)	0 (0–0)

Figures are *n* (column %), mean (SD) or median (IQR).

BMI, body mass index; eGFR, estimated glomerular filtration rate using CKD-EPI formula.

### Prevalence of eGFR <60 mL/min/1.7 m^2^ and incidence of CKD

A total of 23 participants (11 men from Cohort 1 and 12 men from Cohort 2) had an eGFR of <60 mL/min/1.7 m^2^ at baseline and were excluded from the CKD incidence analyses. Of note, five of these participants had an eGFR of >60 mL/min/1.7 m^2^ on repeat testing on at least one occasion during follow-up.

**Figure 2.**
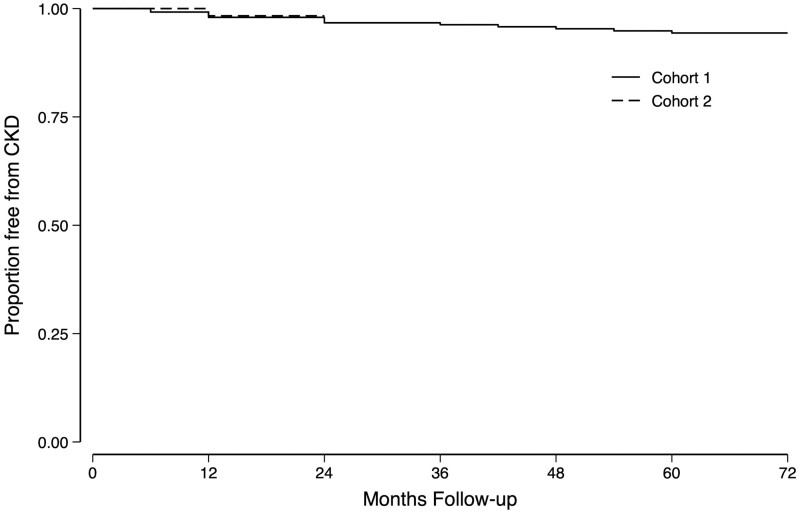
Kaplan–Meier plot of incident chronic kidney disease in the two cohorts. Chronic kidney disease (CKD) is defined as the first of two consecutive estimated glomerular filtration rate (eGFR) measures of <60 mL/min/1.7 m^2^. These measures were >6 months apart by virtue of the study design. No individual classified as having incident CKD reverted to an eGFR of ≥60 mL/min/1.7 m^2^ during the follow-up period

Sixteen participants (13 in Cohort 1 and 3 in Cohort 2), all of whom were men, developed CKD. The overall incidence rate for CKD in males was 0.7%/year (95% CI: 0.5% to 1.2%/year; Figure 2). None of these participants recovered to an eGFR of ≥60 mL/min/1.7 m^2^.

Cumulative duration working in the sugarcane industry [hazard ratio (HR): 1.14, 95% CI: 1.02 to 1.27 per year] and cumulative self-reported symptoms of excess occupational sun exposure (HR: 1.62, 95% CI: 1.09 to 2.40) were risk factors for incident CKD (as were self-reported joint inflammation and back pain; Supplementary Table S2, available as Supplementary data at *IJE* online). Unlike associations with early injury (below), none of cumulative measured or self-reported weight loss, cramps, nausea or vomiting was associated with this outcome.

### Estimates of kidney state in individuals at baseline and at 3–5 years of follow-up using the HMM

The HMM was estimated in those participants with at least four eGFR measures available across the first eight visits in Cohort 1 (*n* = 325, 93%) and all four visits in Cohort 2 (*n* = 318, 76%). The mean of the healthy eGFR distribution was estimated to be 123.8 ± 10.7 mL/min/1.7 m^2^ (and was 15.8 mL/min/1.7 m^2^ higher in pregnancy). Of those with HMM estimates, overall, 64 men (17%) and 5 women (2%) were estimated to be (i.e. a posterior probability of >50%) in an unhealthy kidney state at baseline. By the most recent visit included in the HMM, a further 59 men (15%) and 12 women (5%) were estimated to be in an unhealthy kidney state. Nine participants (including one woman) in an unhealthy state during follow-up were estimated to have reverted to a healthy state at the most recent visit included in the HMM estimation.

**Figure 3.**
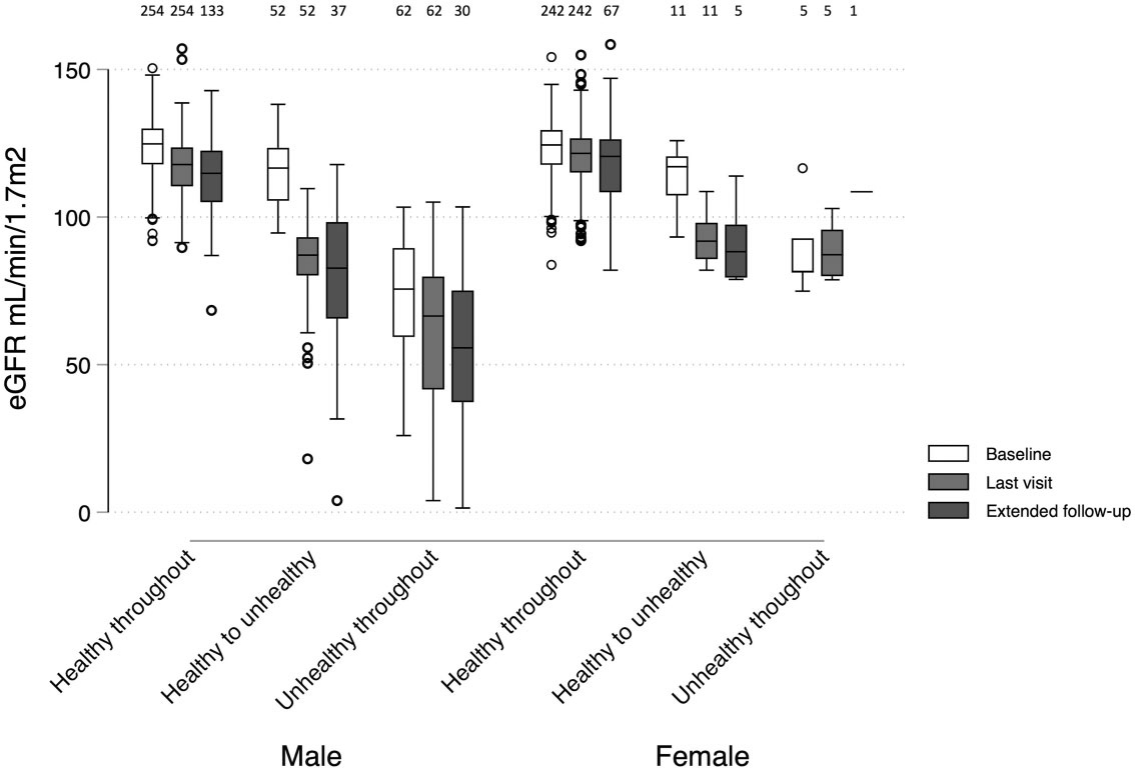
Estimated glomerular filtration rate distributions in men and women at different time points during follow-up stratified by estimated hidden Markov model state at baseline and last visit. Last visit was the latest attended visit with a hidden Markov model estimate (up to Visit 8 for Cohort 1 or Visit 4 for Cohort 2). Participants who reverted from unhealthy to healthy at any point are excluded from this figure (*n* = 9). The number of observations in each group is indicated across the top of the figure. Healthy to unhealthy, healthy at baseline and unhealthy at last visit; extended follow-up, latest of Visits 9 and 10 (Cohort 1 only); eGFR, estimated glomerular filtration rate

No differences were observed in the follow-up duration between the above groups. The eGFR distributions at baseline and last visit stratified by the groups above are summarized in Figure 3 (with individual trajectories and the estimated probabilities of an unhealthy state illustrated in Supplementary Figures S1 and S2, available as Supplementary data at *IJE* online).

### eGFR up to 2 years after the HMM state estimates in Cohort 1

The most recent eGFR (at 84 months or last visit attended in Cohort 1 only) was 115.1 ± 12.4 mL/min/1.73 m^2^ in those who were identified as being in a healthy state throughout based on HMM estimates; 81.3 ± 24.0 mL/min/1.7 m^2^ in those identified as healthy at baseline but being in an unhealthy state based on the most recent HMM estimates; and 58.6 ± 25.8 mL/min/1.7 m^2^ in those participants identified as being in an unhealthy kidney state throughout (Figure 3).

### Episodes of probable departure from ∼eGFR_(healthy)_

**Figure 4.**
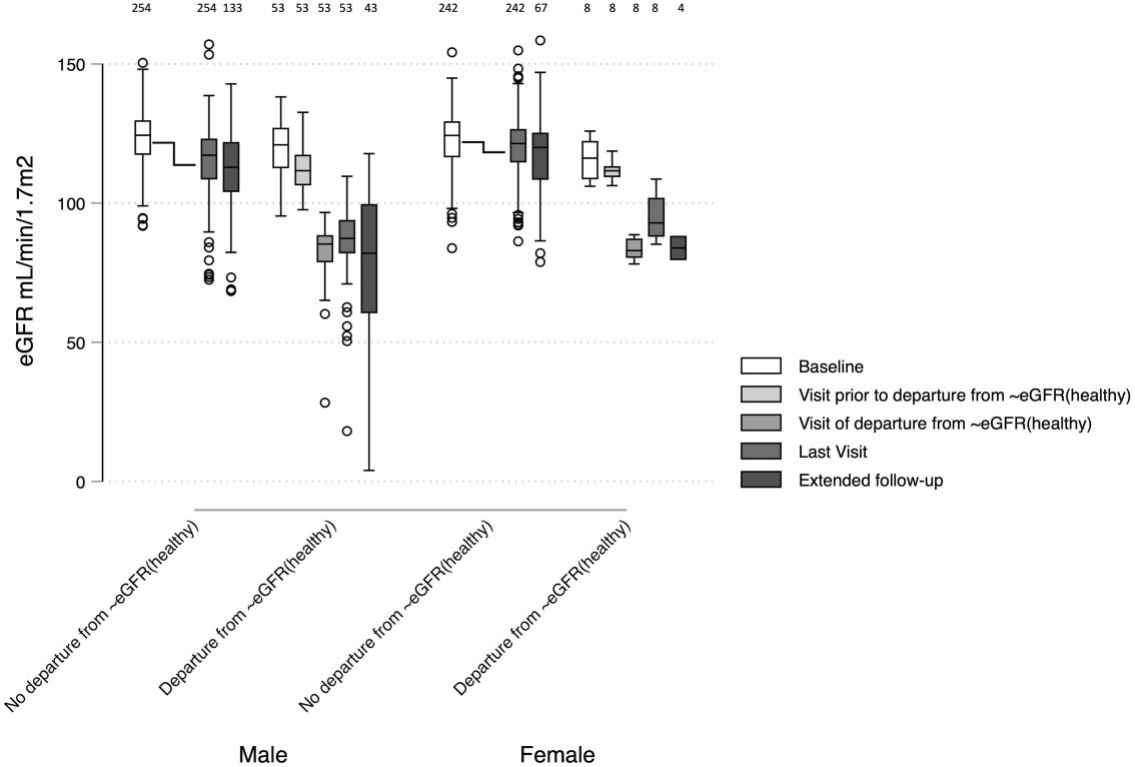
Estimated glomerular filtration rate distributions in men and women at baseline, visit prior to and time of probable departure from the healthy estimated glomerular filtration rate distribution_,_ along with extended follow-up, stratified by those participants who experience departure from the healthy estimated glomerular filtration rate distribution during follow-up. Probable departure from the healthy estimated glomerular filtration rate distribution [departure from ∼eGFR_(healthy)_] was defined as a joint probability of >50% of transitioning from a healthy to unhealthy state since the previous study visit. Visit of departure from ∼eGFR_(healthy)_ varies by participant. The group not experiencing departure from ∼eGFR_(healthy)_ includes only participants estimated to be in a healthy state throughout follow-up. Last visit was the latest attended visit with an hidden Markov model estimate (up to Visit 8 for Cohort 1 or Visit 4 for Cohort 2). Extended follow-up, latest of Visits 9 and 10 (Cohort 1 only). Where an individual’s departure from ∼eGFR_(healthy)_ or pre-departure from ∼eGFR_(healthy)_ visit coincides with the baseline or last visit, the data point is included in both categories for the purposes of this figure. Number of observations in each group is indicated across the top of the figure. eGFR, estimated glomerular filtration rate

Episodes of probable departure from ∼eGFR_(healthy)_ (i.e. a joint probability of >50% of transition between healthy and unhealthy states) were estimated to have occurred at 53 visits in men (2.7% of visits) and 8 visits in women (0.8% of visits) at intervals spread across the follow-up period. Although theoretically possible, no individual sustained more than one probable episode of this event. Individual episodes of probable departure from ∼eGFR_(healthy)_ are shown superimposed on individual eGFR trajectories in Supplementary Figure S1 (available as Supplementary data at *IJE* online) (and estimated probabilities for this outcome by study visit in Supplementary Figure S3, available as Supplementary data at *IJE* online). No differences were observed in the follow-up duration between participants who did and did not sustain probable departure from ∼eGFR_(healthy)_. Episodes of this event were associated with a mean drop in eGFR of 32.8 mL/min/1.7 m^2^ and, similarly to the healthy/unhealthy classification, was associated with a lower eGFR at the most recent visit in the extended follow-up (Figure 4). No discrete episode of departure from ∼eGFR_(healthy)_ could be identified in 10 of the participants who were identified as healthy at baseline but unhealthy at the final HMM estimate [i.e. individuals with a probability of departure from ∼eGFR_(healthy)_ of between 0% and 50% at several time points]. Most participants had normal urinalysis at the visit at which departure from ∼eGFR_(healthy)_ was identified (where available; Supplementary Table S3, available as Supplementary data at *IJE* online).

### Time-updated associations with probability of departure from ∼eGFR_(healthy)_ episodes

In the base model, baseline age was associated with an increased probability [odds ratio (OR): 1.07, 95% CI: 1.01 to 1.13/year] and female sex was associated with a reduced probability (OR: 0.29, 95% CI: 0.26 to 0.63) of departure from ∼eGFR_(healthy)_ episodes after adjustment for community, study visit and follow-up duration. There was no evidence that the outcome occurred more frequently with any individual study visit, increased with follow-up time or in any one community (Supplementary Table S4, available as Supplementary data at *IJE* online).

Time-updated associations with probability of departure from ∼eGFR_(healthy)_ episodes are shown in Tables 2 and 3. When examining occupation(s) since the previous visit, sugarcane was associated with an increased (OR: 1.76, 95% CI: 1.09 to 2.02) and fishing was associated with a reduced (OR: 0.02, 95% 0.01 to 0.03, based on a limited number of participants reporting fishing occupation) probability of these episodes. Self-reported symptoms of excess occupational sun exposure (OR: 1.62, 95% CI: 1.09 to 2.40) was associated with this outcome along with a borderline association with outdoor work.

**Table 2.** Occupational exposures associated with departure from the healthy estimated glomerular filtration rate distribution

Exposure	Odds ratio (95% CI)
Occupation since last visit (worked for a minimum of 2 weeks)^a^	
Sugarcane	**1.76 (1.09–2.82)**
Banana	0.83 (0.34–2.00)
Paid agricultural	0.53 (0.23–1.18)
Unpaid agricultural	1.13 (0.65–1.97)
Commerce	1.3 (0.57–2.96)
Construction	0.6 (0.22–1.58)
Fishing	**0.02 (0.01–0.03)**
Home working	1.24 (0.40–3.80)
Occupational environment (all yes vs no except where stated)	
Work outdoors (regularly or more frequently vs less frequently)	1.58 (0.96–2.59)
Work in a very hot environment (regularly or more frequently vs less frequently)	1.04 (0.68–1.60)
Place to cool off	1.33 (0.32–5.61)
Shade available at workplace	0.38 (0.14–1.07)
Work breaks in the shade	0.47 (0.19–1.18)
Fast pace working	0.98 (0.65–1.48)
Perspiration on arrival at work	0.94 (0.59–1.50)
Heavy lifting at work (regularly or more frequently vs less frequently)	1.00 (0.66–1.52)
Hard physical effort at work in the past week	1.13 (0.59–2.14)
Fainting at work	0.64 (0.12–3.46)
Symptoms of excess sun exposure at work	**1.61 (1.02–2.53)**
Agrochemical use	1.16 (0.72–1.86)

All exposures analysed as time-updated variables. Adjusted for age, sex, study visit and follow-up duration.

a Participants could report more than one occupation at each study visit. Coefficients in bold are where the 95% CI does not include unity.

**Table 3.** Non-occupational exposures associated with departure from the healthy estimated glomerular filtration rate distribution

Exposure	Odds ratio (95% CI)
Social factors (yes vs no unless otherwise stated)	
Piped water source	0.63 (0.35–1.13)
Daily water intake (per litre)	1.09 (0.99–1.20)
Smoking in the past 12 months	1.27 (0.80–2.01)
Alcohol in the past 12 months	1.33 (0.85–2.08)
Other recreational drugs in the past 12 months	1.37 (0.63–2.97)
Self-report of seeing Aristolochia (from photograph)	1.44 (0.52–3.96)
Premature birth	0.97 (0.30–3.18)
Medical factors since last visit (yes vs no unless otherwise stated)	
Mean systolic blood pressure (measured at this visit) (per 10 mmHg)	1.03 (0.87–1.21)
Mean diastolic blood pressure (measured at this visit) (per 10 mmHg)	1.20 (0.99–1.47)
Weight loss of >2.5 kg (measured since last visit)	**1.95 (1.18–3.24)**
Diagnosis of urinary tract infection	1.35 (0.69–2.65)
Diagnosis of kidney stones	1.00 (0.19–5.17)
Diagnosis of chronic kidney disease	2.14 (0.56–8.21)
Diagnosis of Chikungunya^a^	2.25 (0.90–5.61)
Diagnosis of dengue^a^	**0.10 (0.03–0.35)**
Diagnosis of or medication for high blood pressure	1.39 (0.37–5.28)
NSAIDs regularly or more frequently	**2.10 (1.01–4.37)**
Paracetamol regularly or more frequently	0.65 (0.19–2.22)
Any antibiotic injections	1.51 (0.42–5.52)
Symptoms since last visit (more frequent than ‘never or almost never’ vs ‘never or almost never’ unless otherwise stated)	
Any back pain	1.11 (0.74–1.67)
Unintentional weight loss	**1.55 (1.00–2.39)**
Dry mouth	1.11 (0.62–2.01)
Dysuria	0.84 (0.54–1.30)
Oliguria	1.16 (0.70–1.93)
Tachycardia	1.39 (0.80–2.43)
Cramps	**2.25 (1.35–3.73)**
Headache more than once a month	1.44 (0.91–2.28)
Fever	1.56 (0.99–2.47)
Tremor	0.93 (0.26–3.28)
Joint inflammation	0.47 (0.11–1.93)
Nausea	**1.86 (1.07–3.25)**
Dyspnoea	1.75 (0.72–4.22)
Pre-syncope	1.29 (0.61–2.73)
Syncope	2.82 (0.81–9.85)
Diarrhoea	0.65 (0.26–1.66)
Vomiting	**2.26 (1.17–4.35)**
Nosebleed	0.90 (0.14–5.61)
Dyspepsia	1.29 (0.71–2.33)
Earache	0.98 (0.30–3.17)
Confusion	0.89 (0.30–2.66)
Tiredness	1.20 (0.72–1.99)

All exposures analysed as time-updated variables. Adjusted for age, sex, study visit and follow-up duration.

a Restricted analysis as data not available for Visits 1–3. Coefficients in bold are where the 95% CI does not include unity.

NSAID, non-steroidal anti-inflammatory drug.

A strong negative association was seen between diagnosis with dengue and departure from ∼eGFR_(healthy)_ episodes, although only 20 individuals reported this diagnosis and data were only available for 7 of the 10 study visits so this finding should be interpreted with caution. Measured weight loss of >2.5 kg (OR: 1.95, 95% CI: 1.18 to 3.24) was associated with probability of these episodes, as were reports of regular or more frequent non-steroidal anti-inflammatory drug (NSAID) use (OR: 2.10, 95% CI: 1.01 to 4.37) along with a borderline association with diastolic blood pressure. There was also an association between increased probability of this outcome and self-report of unintentional weight loss (OR: 1.55, 95% CI: 1.00 to 2.39), increased frequency of cramps (OR: 2.25, 95% CI: 1.35 to 3.73), nausea (OR: 1.86, 95% CI: 1.07 to 3.25) and vomiting (OR: 2.26, 95% CI: 1.17 to 4.35) along with a borderline association with fever.

In multivariable analyses, measured weight loss and cramps remained independently associated with probability of departure from ∼eGFR_(healthy)_ episodes after adjustment for sugarcane work and surrogates for heat stress (Supplementary Table S5, available as Supplementary data at *IJE* online). Stratification of excess occupational sun exposure, cramps and weight loss by sex and season (Supplementary Table S6, available as Supplementary data at *IJE* online) suggested that the latter two exposures were not primarily work-related (i.e. not more common in both men and post-harvest when examined biannually).

## Discussion and conclusion

This study currently represents the only population-based longitudinal study in those at risk of MeN reported to date. The original cohort has now been followed up for 7 years and the close to 1%/year incidence of CKD amongst men confirms the huge burden of kidney disease in this population, which is probably at least 25 times that seen in the USA in similar age groups.^17^ Cumulative duration spent working in the sugarcane industry was a risk factor for established CKD consistently with multiple previous cross-sectional studies.^7^^,^^18^^,^^19^ Although this study does not provide specific insight, there is a body of work suggesting that it is a physically demanding occupation undertaken in often unmitigated environmental heat,^20^ the importance of which is further supported by the association with self-reported excess occupational sun exposure.

We used the HMM to identify the time point of departure from ∼eGFR_(healthy)_ and demonstrated that a decline from baseline over the previous 6–12 months often leads to a sustained reduction in eGFR, suggesting either an acute kidney injury (AKI) with non-recovery or a form of sub-acute/early chronic kidney injury. This is important for studies of MeN as there is typically no haematuria or proteinuria, which are useful markers for the onset of other renal diseases. Although similar drops in eGFR have been described previously in occupational studies,^4^^,^^5^ this is the first description of empirically determined early injury at the population level. The key advantages of the HMM approach over analyses using other renal outcomes is the estimation of a probability of departure from ∼eGFR_(healthy)_ for each participant at every study visit, whilst accounting for an individual’s entire renal trajectory over the follow-up period. For example, HMM compares favourably to analyses examining associations using a binary threshold (e.g. a 30% decline in eGFR) where misclassification will occur both with potentially important sub-threshold drops in eGFR and where there is a rapid return to the baseline (suggesting reversible AKI, not early CKD). Similarly, the HMM approach has advantages over an eGFR slope outcome analysis calculated over multiple visits, which, by design, cannot identify the time point of the earliest kidney injury and may predominantly demonstrate associations with exacerbating rather than initiating factors.

The association between probability of departure from ∼eGFR_(healthy)_ episodes and NSAIDs provides internal validation for the methodological approach as these drugs are known to cause kidney injury. Similarly, the association of symptoms of excess occupational sun exposure and this outcome was not unexpected given previously reported findings.^5^ Whether the associations with occupation(s) (sugarcane and fishing) are also surrogates for greater or lower heat-related exposure remains unclear.

The association of probability of departure from ∼eGFR_(healthy)_ episodes with measured and self-reported weight loss, along with cramps, nausea and vomiting since the last study visit, is novel and raises key questions as to the cause of this syndrome. It is unlikely that these findings are a consequence of the reduction in eGFR per se as changes in clearance at these well preserved eGFRs would be expected to be asymptomatic. It is possible that this syndrome reflects dehydration or heat illness not captured by other questionnaire responses aimed at these exposures. However, reports of cramps and weight loss at similar frequencies in men and women, as well as the finding that associations between departure from ∼eGFR_(healthy)_ and these same factors remained quantitatively similar in multivariable models, make this unlikely. Therefore, the possibility of this syndrome representing a response to exposure to infection^21^ or non-infective xenobiotic^22^ should be considered.

We acknowledge that the evidence that these episodes of departure from ∼eGFR_(healthy)_ represent the initial stages of MeN is not definitive. However, the sustained drop in kidney function at relatively preserved eGFR, which is common at the population level where disease is prevalent, support the idea that these episodes reflect an early manifestation of MeN and further interrogation of these episodes and the associated symptoms/weight loss may provide key aetiological insight.

### Limitations

Despite the burden of disease, the number of new CKD events remains relatively low, which limits the power to detect weaker associations with this outcome. Loss to follow-up, although low, was highest among men, who in turn were more likely to experience kidney injury, potentially leading to an underestimation of both outcomes.

Although the HMM approach is probably the optimal method available to explore the earliest stages of MeN, there likely remains residual misclassification, with some departure from ∼eGFR_(healthy)_ episodes representing exacerbation of pre-existing kidney injury and others disease-unrelated episodes of AKI (e.g. due to NSAIDs). This highlights the importance of identifying a novel robust (urinary) biomarker to detect early MeN. Furthermore, our assessments were made annually or biannually, meaning that the analysis windows were wide, limiting the precision to examine co-incident exposures with the earliest changes in kidney function.

Lastly, almost all exposure assessments relied on self-report, which are also prone to misclassification and may be gender- or otherwise context-specific. However, this misclassification is likely to lead to an underestimation of associations.

### Conclusion

This representative prospective study confirms the burden of kidney disease in Pacific Coast Central America with remarkably high CKD incidence rates in young men and confirms that sugarcane work and other surrogates of heat stress are risk factors for established disease. We also identified changes in eGFR reflecting the earliest departure from a state of kidney health. These early changes occur in both men and women, although more commonly in men. Factors associated with this early injury include cramps and weight loss, and the findings support the possibility of separate initiating and exacerbating factors in the pathogenesis of MeN. The initiating factor(s) remain(s) unknown and, whilst initiatives to uncover these exposures are key, interventions to reduce the impact of exacerbating factors should be pursued in the meantime.

## Ethics approval

Approval for this work was received from the institutional review boards at Universidad Nacional Autonoma de Nicaragua, Leon (Acta No.116, Ano 2014; Acta No.71, Ano 2018), the London School of Hygiene and Tropical Medicine (Ref: 8643) and University College London (Ref: 14175).

## Supplementary Material

dyad151_Supplementary_Data

## Data Availability

The original data underlying this article cannot be shared publicly due to the privacy of individuals who participated in the study. Non-identifiable or summary data will be shared on reasonable request to the study steering group through the corresponding author.
