# Correction to: Population-level detection of early loss of kidney function: 7-year follow-up of a young adult cohort at risk of Mesoamerican nephropathy

**DOI:** 10.1093/ije/dyad163

**Published:** 2023-11-29

This is a correction to: Marvin Gonzalez-Quiroz, Brianna Heggeseth, Armando Camacho, Amin Oomatia, Ali M Al-Rashed, Yixuan Zhang, Alexander McCreight, Nicholas Jewell, Aurora Aragon, Dorothea Nitsch, Neil Pearce, Ben Caplin, Population-level detection of early loss of kidney function: 7-year follow-up of a young adult cohort at risk of Mesoamerican nephropathy, *International Journal of Epidemiology*, 2023, dyad151, https://doi.org/10.1093/ije/dyad151

In the originally published version of this manuscript, there was a typographical error in author Nicholas Jewell’s name.

This has been corrected.

