# Supplementary material for: Population-level detection of early loss of kidney function: 7-year follow-up of a young adult cohort at risk of Mesoamerican nephropathy
Source: Int J Epidemiol. 2023 Oct 31;53(1):dyad151. doi: 10.1093/ije/dyad151 (PMC10859140; doi:10.1093/ije/dyad151)
Supplement: dyad151_Supplementary_Data [file dyad151_supplementary_data.docx]

Supplementary Appendix

**Population-level detection of early loss of kidney function: 7-year follow-up of a young adult cohort at risk of Mesoamerican Nephropathy**

Marvin Gonzalez-Quiroz, Brianna Heggeseth, Armando Camacho, Amin Oomatia, Ali Al-Rashed, Yixuan Zhang, Alexander McCreight, Nicholas Jewel, Aurora Aragon, Dorothea Nitsch, Neil Pearce, Ben Caplin

Supplementary Methods………………………………………………………………………..……….2

[Supplementary Table S1– Median proportion of follow-up study visits that each exposure was reported stratified by Cohort and Sex. 5](#_Toc148015582)

[Supplementary Table S2 *-* Associations with incident chronic kidney disease in males. 6](#_Toc148015583)

[Supplementary Table S3 – Base fractional regression model for departure from the estimated glomerular filtration rate distribution. 7](#_Toc148015584)

[Supplementary Table S4 - Urinalysis findings at study visits stratified by Hidden Markov Model estimates of kidney status. 8](#_Toc148015585)

[Supplementary Table S5 –Exploratory multivariable models of associations with departure from the healthy estimated glomerular filtration rate distribution. 9](#_Toc148015586)

[Supplementary Table S6 – Exploration of proportion reporting cramps, occupational sun exposure and measured weight loss by season and sex 10](#_Toc148015587)

Supplementary Figure S1 – Spaghetti plots of individual estimated glomerular filtration rate trajectories stratified by healthy-unhealthy status and sex in Cohort 1and 2. 11

Supplementary Figure S2 - Distribution of estimated posterior probabilities of unhealthy state from Hidden Markov Model by study visit in Cohort 1 and 2.. 13

Supplementary Figure S3 – Distribution of estimated probability of departure from the estimated glomerular filtration rate derived from the Hidden Markov Model by study visit in Cohort 1 and 2... 14

Supplementary Figure S4 - QQ plots of residuals and random effects parameters from linear mixed model………………………………………………………...………………………………………...15

Supplementary Methods

*Study population and study procedures*

Participants described in this report were invited to take part in two phases (Cohort 1 and 2) from 11 rural communities in Leon and Chinandega departments, Nicaragua. Inclusion criteria for all cohorts were age 18-30 years and absence of self-reported kidney disease, diabetes, or hypertension. The 351 participants from Cohort 1 (enrolled 2014/15) are from nine communities and were recruited at a male-to-female ratio of 3:1. Cohort 2 consists of 420 participants with a male-to-female ratio 1:1 enrolled in 2018 from the existing communities and two new communities. This change was instituted to increase the numbers of women in the study as it became clear that not only men, as initially suspected, but also women appeared to be affected by MeN (albeit at lower incidence).

Questionnaire data, clinical measurements and biological samples were collected at baseline and then either 6-monthly (Cohort 1 for the first two years) or annually, initially by hand and then using tablet-based data collection software tools (Open Data Kit^1^). Participants were asked to respond to questions on demography, occupational history and current job, occupational exposures, lifestyle factors, medications, and symptoms. Except at baseline (where detailed occupational histories were taken and symptoms were assessed over the last 6-months), questions asked whether participants had experienced the relevant exposure since the last study visit (unless indicated otherwise). Body weight and height were measured, along with blood pressure (BP) and heart rate (mean of three measurements), in a sitting position using a calibrated digital sphygmomanometer (Omron, Kyoto, Japan) after five minutes of quiet seated rest.

Participants provided spot urine samples which was aliquoted and placed in ice in the field immediately. Blood samples were collected and centrifuged to separate serum, aliquoted, and immediately stored on ice. All samples were collected prior to the workday where possible and transported in ice to the laboratory at UNAN-León, where aliquots were frozen at -20^o^C. A local serum creatinine measurement was performed to provide feedback on eGFR to study participants. Also, from Visit 7 point of care testing was performed for urine specific gravity, pH, protein, blood, leucocytes, nitrite, and glucose.

### Laboratory procedures

Serum creatinine for the purposes of this analysis were measured in three batches by the Clinical Trials Services Unit, University of Oxford and Health Services Laboratory, London, using quality control referenced to international standards. To account for batch-to-batch variation ~100 duplicate samples across the range of creatinine were remeasured at each assay run and a correction factor applied. eGFR was calculated using the original (2009) CKD-EPI formula^2^.

### Basic statistical methods

Descriptive statistics are presented as frequencies and percent (%), means$+/-$ standard deviation (SD), or medians and interquartile range (IQR) as appropriate. For non-continuous exposures, including work environment and symptoms, variables were recoded as dichotomous, i.e., above or below the median which typically (and except where stated otherwise) classified participants as experiencing the exposure ‘never or almost never’ versus more frequently. Weight loss was defined as a drop in weight from the previous study visit of >2.5kg.

CKD (stage 3-5) free survival was estimated using the Kaplan-Meier method with outcome defined as the time of the first of two consecutive eGFR measures <60mL/min/1.7m^2^ (which by virtue of study design were >6 months apart), with the last visit (to avoid immortal time) and deaths censored.

We also explored the possibility of modelling eGFR decline over time using a linear model in the study population. eGFR values were nested within participants in a linear mixed model including a random intercept and slope for follow-up time. Models were adjusted for age and sex. However, examination of the distribution of random effects parameters suggested these models fit the data poorly (Supplementary Figure 4). This, combined with our aim of studying the earliest departure from a state of kidney health meant we went on to explore a novel kidney outcome using Hidden-Markov modelling.

### Hidden Markov modelling

A continuous-time Hidden Markov model (HMM) was used to study development of disease based on eGFR measurements over time. Two underlying latent kidney states, healthy and unhealthy, were assumed, with associated eGFR distributions. We approximated the eGFR distributions for both kidney function states by assuming Gaussian distributions for computational accessibility and simplicity. While unhealthy eGFR distributions will be left-skewed, the model should nonetheless provide a robust approximation to capture the probabilities of transitioning from a healthy to unhealthy kidney state.

A range of starting values allowing the detection of both early and established disease were examined and optimal model fit (as determined by the Akaike information criterion) was achieved with a mean eGFR 75 mL/min/1.73m^2^ and standard deviation of 15 mL/min/1.73m^2^ for the unhealthy state. The centre and scale of the eGFR distribution for the healthy state was estimated empirically, while allowing values during pregnancy to deviate from these overall distributions.

The chance of switching between states (which could occur in both directions), known as the *transition probabilities*, were modelled with an empirically derived indicator of greater than a 10% decline in eGFR from baseline to account for individual variation in eGFR within the states. Therefore, if an individual has an eGFR of 100 mL/min/1.73m^2^ but started at baseline with an eGFR of 120 mL/min/1.73m^2^, it is more likely that this individual has transitioned to the unhealthy state as compared to if that individual had started at baseline with an eGFR of 105 mL/min/1.73m^2^. Various thresholds (5% decline, 10% decline, 5 mL/min/1.73m^2^ decline, 10 mL/min/1.73m^2^ decline were considered and we used the threshold with the best model fit (highest likelihood).

Importantly, the HMM did not just identify all episodes of reversible acute kidney injury, as there were also isolated measures of eGFR<90mL/min/1.73m^2^ in 10 individuals with otherwise preserved kidney function where participants remained classified as healthy throughout the follow-up period.

The HMM was fit using the msm R package (version 1.6.9) ^3^ across both cohorts simultaneously and the final two visits for Cohort 1 were omitted from the HMM estimates so the medium-term consequence of the HMM model could be explored.

Based on the fit HMM, the posterior probability of being in a healthy or unhealthy state and the joint probability of transition from healthy to unhealthy were estimated. This transition, or departure from ~eGFR_(healthy)_, was defined as the estimated joint probability that an individual is in an unhealthy kidney state at the current visit having transitioned from a healthy state at the previous study visit. These probabilities are estimated at each observation time and based on the entire longitudinal trajectory of eGFR measures.

### Risk factor analysis

Cohorts were combined and exposure variables (except age, systolic and diastolic BP, and cumulative duration working in each occupation) were dichotomized. Potential associations with risk factors for incident CKD were explored in males only using a Cox-proportional hazards model with associations adjusted for age with the proportional hazards’ assumption tested by examining Schoenfeld residuals.

Once kidney state and departure from ~eGFR_(healthy)_ probabilities were estimated with the HMM, risk factors were interrogated using a fractional logit model with the outcome as the probability of departure from ~eGFR_(healthy)_ at each study visit, including observations where an individual was estimated to be in a healthy state at the previous visit. Clustered robust standard errors were used to account for within individual correlation. Fractional regression exposure models were *a priori* adjusted for baseline age, sex, study visit and follow-up duration in all exposure analyses. Associations were reported where the 95% confidence interval (95%CI) for the odds ratio (OR) did not include one.

References

1. Hartung C, Lerer A, Anokwa Y, Tseng C, Brunette W, Borriello G. Open data kit: tools to build information services for developing regions. Proceedings of the 4th ACM/IEEE International Conference on Information and Communication Technologies and Development. London, United Kingdom: Association for Computing Machinery; 2010:Article 18.

2. Levey AS, Stevens LA, Schmid CH, et al. A new equation to estimate glomerular filtration rate. Ann Intern Med 2009;150(9):604-12. DOI: 10.7326/0003-4819-150-9-200905050-00006.

3. Jackson C. Multi-State Models for Panel Data: The msm Package for R. Journal of Statistical Software 2011;38(8):1 - 28. DOI: 10.18637/jss.v038.i08.

**Supplementary Table S1– Median proportion of follow-up study visits that each exposure was reported stratified by Cohort and Sex.**

|  | Cohort | | | | | | | | | | | |
| --- | --- | --- | --- | --- | --- | --- | --- | --- | --- | --- | --- | --- |
|  | 1 (up to 9 visits) | | | | | | 2 (up to 3 visits) | | | | | |
|  | Sex | | | | | | Sex | | | | | |
|  | Male | | | Female | | | Male | | | Female | | |
| *Occupation since last visit (worked for a minimum of 2 weeks)** |  |  |  |  |  |  |  |  |  |  |  |  |
| Sugarcane work | 0.4 | (0.1 to | 0.7) | 0.0 | (0.0 to | 0.0) | 0.3 | (0.0 to | 0.7) | 0.0 | (0.0 to | 0.0) |
| Banana work | 0.0 | (0.0 to | 0.0) | 0.0 | (0.0 to | 0.0) | 0.0 | (0.0 to | 0.0) | 0.0 | (0.0 to | 0.0) |
| Paid agricultural work | 0.1 | (0.0 to | 0.2) | 0.0 | (0.0 to | 0.0) | 0.0 | (0.0 to | 0.5) | 0.0 | (0.0 to | 0.0) |
| Unpaid agricultural work | 0.1 | (0.0 to | 0.3) | 0.0 | (0.0 to | 0.0) | 0.0 | (0.0 to | 0.3) | 0.0 | (0.0 to | 0.0) |
| Commerce work | 0.0 | (0.0 to | 0.0) | 0.0 | (0.0 to | 0.1) | 0.0 | (0.0 to | 0.0) | 0.0 | (0.0 to | 0.0) |
| Construction work | 0.0 | (0.0 to | 0.1) | 0.0 | (0.0 to | 0.0) | 0.0 | (0.0 to | 0.0) | 0.0 | (0.0 to | 0.0) |
| Fishing work | 0.0 | (0.0 to | 0.0) | 0.0 | (0.0 to | 0.0) | 0.0 | (0.0 to | 0.0) | 0.0 | (0.0 to | 0.0) |
| Home work | 0.0 | (0.0 to | 0.0) | 0.8 | (0.6 to | 1.0) | 0.0 | (0.0 to | 0.0) | 1.0 | (0.3 to | 1.0) |
| *Work environment* |  |  |  |  |  |  |  |  |  |  |  |  |
| Working outside | 0.8 | (0.6 to | 0.9) | 0.1 | (0.0 to | 0.2) | 0.7 | (0.5 to | 0.8) | 0.0 | (0.0 to | 0.2) |
| Working in a hot environment | 0.7 | (0.5 to | 0.8) | 0.3 | (0.2 to | 0.4) | 0.5 | (0.5 to | 0.8) | 0.2 | (0.0 to | 0.5) |
| Physical hard work | 0.1 | (0.0 to | 0.1) | 0.0 | (0.0 to | 0.1) | 0.0 | (0.0 to | 0.0) | 0.0 | (0.0 to | 0.0) |
| Lifting heavy objects | 0.4 | (0.2 to | 0.5) | 0.2 | (0.1 to | 0.4) | 0.2 | (0.0 to | 0.5) | 0.2 | (0.0 to | 0.2) |
| Opportunity to cool off | 0.7 | (0.5 to | 0.8) | 0.3 | (0.2 to | 0.4) | 0.5 | (0.5 to | 0.8) | 0.2 | (0.0 to | 0.5) |
| Shade availability at work | 0.9 | (0.8 to | 0.9) | 0.9 | (0.9 to | 0.9) | 0.8 | (0.7 to | 0.8) | 0.8 | (0.8 to | 0.8) |
| Taking breaks in the shade | 0.9 | (0.8 to | 0.9) | 0.9 | (0.9 to | 0.9) | 0.8 | (0.7 to | 0.8) | 0.8 | (0.8 to | 0.8) |
| Fainting at work | 0.0 | (0.0 to | 0.0) | 0.0 | (0.0 to | 0.0) | 0.0 | (0.0 to | 0.0) | 0.0 | (0.0 to | 0.0) |
| Agrichemical use | 0.4 | (0.2 to | 0.7) | 0.0 | (0.0 to | 0.1) | 0.2 | (0.0 to | 0.5) | 0.0 | (0.0 to | 0.0) |
| Working at a fast pace | 0.2 | (0.1 to | 0.4) | 0.2 | (0.0 to | 0.3) | 0.2 | (0.0 to | 0.3) | 0.0 | (0.0 to | 0.2) |
| Being sweaty on arrival at work | 0.2 | (0.1 to | 0.5) | 0.0 | (0.0 to | 0.1) | 0.3 | (0.0 to | 0.7) | 0.0 | (0.0 to | 0.3) |
| Symptoms of excess occupational sun exposure | 0.2 | (0.0 to | 0.3) | 0.0 | (0.0 to | 0.1) | 0.0 | (0.0 to | 0.2) | 0.0 | (0.0 to | 0.0) |
| *Medical factors* |  |  |  |  |  |  |  |  |  |  |  |  |
| Regular NSAID use | 0.0 | (0.0 to | 0.0) | 0.0 | (0.0 to | 0.1) | 0.0 | (0.0 to | 0.0) | 0.0 | (0.0 to | 0.0) |
| Regular paracetamol use | 0.0 | (0.0 to | 0.0) | 0.0 | (0.0 to | 0.1) | 0.0 | (0.0 to | 0.0) | 0.0 | (0.0 to | 0.0) |
| Antibiotic use | 0.0 | (0.0 to | 0.0) | 0.0 | (0.0 to | 0.0) | 0.0 | (0.0 to | 0.0) | 0.0 | (0.0 to | 0.0) |
| Measured weight loss >2.5kg | 0.1 | (0.0 to | 0.2) | 0.1 | (0.0 to | 0.2) | 0.0 | (0.0 to | 0.2) | 0.0 | (0.0 to | 0.2) |
| *Symptoms* |  |  |  |  |  |  |  |  |  |  |  |  |
| Back pain | 0.4 | (0.1 to | 0.6) | 0.5 | (0.3 to | 0.7) | 0.2 | (0.0 to | 0.5) | 0.5 | (0.2 to | 0.7) |
| Unintentional weight loss | 0.2 | (0.0 to | 0.3) | 0.2 | (0.1 to | 0.4) | 0.0 | (0.0 to | 0.2) | 0.0 | (0.0 to | 0.2) |
| Dry mouth | 0.0 | (0.0 to | 0.2) | 0.0 | (0.0 to | 0.1) | 0.0 | (0.0 to | 0.0) | 0.0 | (0.0 to | 0.0) |
| Dysuria | 0.2 | (0.0 to | 0.3) | 0.2 | (0.0 to | 0.4) | 0.0 | (0.0 to | 0.2) | 0.2 | (0.0 to | 0.5) |
| Oliguria | 0.1 | (0.0 to | 0.2) | 0.1 | (0.0 to | 0.3) | 0.0 | (0.0 to | 0.2) | 0.0 | (0.0 to | 0.2) |
| Tachycardia | 0.0 | (0.0 to | 0.2) | 0.0 | (0.0 to | 0.2) | 0.0 | (0.0 to | 0.2) | 0.0 | (0.0 to | 0.2) |
| Cramps | 0.0 | (0.0 to | 0.1) | 0.1 | (0.0 to | 0.1) | 0.0 | (0.0 to | 0.2) | 0.0 | (0.0 to | 0.2) |
| Headache | 0.1 | (0.0 to | 0.2) | 0.2 | (0.1 to | 0.3) | 0.0 | (0.0 to | 0.2) | 0.2 | (0.0 to | 0.5) |
| Fever | 0.1 | (0.0 to | 0.3) | 0.1 | (0.0 to | 0.2) | 0.2 | (0.0 to | 0.2) | 0.2 | (0.0 to | 0.2) |
| Tremor | 0.0 | (0.0 to | 0.0) | 0.0 | (0.0 to | 0.0) | 0.0 | (0.0 to | 0.0) | 0.0 | (0.0 to | 0.0) |
| Inflammation | 0.0 | (0.0 to | 0.0) | 0.0 | (0.0 to | 0.0) | 0.0 | (0.0 to | 0.0) | 0.0 | (0.0 to | 0.0) |
| Nausea | 0.0 | (0.0 to | 0.1) | 0.0 | (0.0 to | 0.1) | 0.0 | (0.0 to | 0.0) | 0.0 | (0.0 to | 0.2) |
| Dyspnoea | 0.0 | (0.0 to | 0.0) | 0.0 | (0.0 to | 0.0) | 0.0 | (0.0 to | 0.0) | 0.0 | (0.0 to | 0.0) |
| Presyncope | 0.0 | (0.0 to | 0.1) | 0.0 | (0.0 to | 0.1) | 0.0 | (0.0 to | 0.0) | 0.0 | (0.0 to | 0.2) |
| Syncope | 0.0 | (0.0 to | 0.0) | 0.0 | (0.0 to | 0.0) | 0.0 | (0.0 to | 0.0) | 0.0 | (0.0 to | 0.0) |
| Diarrhoea | 0.0 | (0.0 to | 0.1) | 0.0 | (0.0 to | 0.0) | 0.0 | (0.0 to | 0.0) | 0.0 | (0.0 to | 0.0) |
| Vomiting | 0.0 | (0.0 to | 0.1) | 0.0 | (0.0 to | 0.0) | 0.0 | (0.0 to | 0.0) | 0.0 | (0.0 to | 0.0) |
| Nosebleed | 0.0 | (0.0 to | 0.0) | 0.0 | (0.0 to | 0.0) | 0.0 | (0.0 to | 0.0) | 0.0 | (0.0 to | 0.0) |
| Dyspepsia | 0.0 | (0.0 to | 0.1) | 0.1 | (0.0 to | 0.2) | 0.0 | (0.0 to | 0.2) | 0.0 | (0.0 to | 0.2) |
| Earache | 0.0 | (0.0 to | 0.0) | 0.0 | (0.0 to | 0.0) | 0.0 | (0.0 to | 0.0) | 0.0 | (0.0 to | 0.0) |
| Confusion | 0.0 | (0.0 to | 0.0) | 0.0 | (0.0 to | 0.0) | 0.0 | (0.0 to | 0.0) | 0.0 | (0.0 to | 0.0) |
| Tiredness | 0.1 | (0.0 to | 0.2) | 0.1 | (0.0 to | 0.2) | 0.0 | (0.0 to | 0.2) | 0.2 | (0.0 to | 0.3) |

NSAID: non-steroidal anti-inflammatory drug. Median (IQR). Does not include baseline visit. *Participants could report more than one occupation at each study visit.

**Supplementary Table S2 *-* Associations with incident chronic kidney disease in males.**

|  | Hazard Ratio | (95% Confidence Interval) |
| --- | --- | --- |
| Piped water source, yes versus no | 0.89 | (0.50 to 1.59) |
| Alcohol use | 1.15 | (0.87 to 1.53) |
| Smoking | 1.21 | (0.94 to 1.56) |
| Cumulative reports of drug use | 1.31 | (0.92 to 1.88) |
| Premature birth, yes versus no | 0.28 | (0.06 to 1.22) |
| **Cumulative years worked in sugarcane, per year** | **1.14** | **(1.02 to 1.27)** |
| Cumulative years worked in banana, per year | 0.99 | (0.74 to 1.32) |
| Cumulative years worked in other paid agriculture, per year | 1.04 | (0.87 to 1.24) |
| Cumulative years worked in unpaid agriculture, per year | 1.00 | (0.89 to 1.13) |
| Cumulative years worked in commerce, per year | 1.12 | (0.75 to 1.66) |
| Cumulative years worked in construction, per year | 0.21 | (0.01 to 4.06) |
| Cumulative reports of working outside | 1.27 | (0.85 to 1.92) |
| Cumulative reports of working in a hot environment | 1.14 | (0.74 to 1.75) |
| Cumulative reports of opportunity to cool off | 1.10 | (0.72 to 1.66) |
| Cumulative reports of shade availability at work | 1.21 | (0.63 to 2.34) |
| Cumulative reports of taking breaks in the shade | 1.30 | (0.67 to 2.52) |
| Cumulative reports of working at a fast pace | 1.19 | (0.87 to 1.62) |
| Cumulative reports of being sweaty on arrival at work | 1.22 | (0.84 to 1.75) |
| Cumulative reports of lifting heavy objects | 1.08 | (0.71 to 1.66) |
| Cumulative reports of physical hard work | 1.01 | (0.52 to 1.97) |
| Cumulative reports of fainting at work | 1.89 | (0.27 to 13.00) |
| **Cumulative reports of symptoms of excessive occupational sun exposure** | **1.62** | **(1.09 to 2.40)** |
| Cumulative reports of agrichemical use | 1.29 | (0.86 to 1.93) |
| Time updated systolic BP | 1.13 | (0.71 to 1.81) |
| Time updated diastolic BP | 1.26 | (0.70 to 2.28) |
| Cumulative reports of regular NSAID use | 1.52 | (0.75 to 3.06) |
| Cumulative reports of regular paracetamol use | 0.82 | (0.21 to 3.27) |
| Cumulative reports of antibiotic use | 0.41 | (0.06 to 2.98) |
| Cumulative measured weight loss >2.5kg/visit | 0.59 | (0.20 to 1.70) |
| **Cumulative reports of back pain** | **1.44** | **(1.03 to 2.00)** |
| Cumulative reports of unintentional weight loss | 1.05 | (0.68 to 1.62) |
| Cumulative reports of dry mouth | 1.10 | (0.66 to 1.84) |
| Cumulative reports of dysuria | 0.65 | (0.37 to 1.14) |
| Cumulative reports of oliguria | 0.69 | (0.34 to 1.41) |
| Cumulative reports of tachycardia | 1.21 | (0.68 to 2.17) |
| Cumulative reports of cramps | 0.89 | (0.46 to 1.70) |
| Cumulative reports of headache | 0.82 | (0.44 to 1.54) |
| Cumulative reports of fever | 1.31 | (0.85 to 2.03) |
| Cumulative reports of tremor | 1.43 | (0.55 to 3.76) |
| **Cumulative reports of inflammation** | **2.01** | **(1.15 to 3.52)** |
| Cumulative reports of nausea | 1.07 | (0.59 to 1.94) |
| Cumulative reports of dyspnoea | 1.27 | (0.46 to 3.50) |
| Cumulative reports of presyncope | 1.50 | (0.77 to 2.93) |
| Cumulative reports of syncope | 2.22 | (0.39 to 12.50) |
| Cumulative reports of diarrhoea | 0.70 | (0.17 to 2.99) |
| Cumulative reports of vomiting | 1.32 | (0.68 to 2.58) |

Adjusted for age. NSAID: non-steroidal anti-inflammatory drug; BP: blood pressure; Except where stated, all associations with cumulative number of study visits where exposure reported (as either, ‘yes’ versus ‘no’ or more frequently than 'never or almost never' versus 'never or almost never', except headache which was dichotomised as more or less than monthly). Coefficients in **bold** where 95% CI do not include unity.

**Supplementary Table S3 – Base fractional regression model for departure from the estimated glomerular filtration rate distribution.**

|  | Odds Ratio | (95% Confidence Interval) |
| --- | --- | --- |
| **Age, each year above 23 years old** | **1.07** | **(1.01 to 1.13)** |
| **Female** | **0.29** | **(0.18 to 0.47)** |
| Years follow-up | 1.04 | (0.82 to 1.32) |
| Visit* |  |  |
| 2 | Reference |  |
| 3 | 0.64 | (0.29 to 1.38) |
| 4 | 1.06 | (0.48 to 2.31) |
| 5 | 0.40 | (0.13 to 1.18) |
| 6 | 1.31 | (0.51 to 3.32) |
| 7 | 0.48 | (0.16 to 1.43) |
| 8 | 0.86 | (0.30 to 2.46) |
| 9 | 0.88 | (0.32 to 2.40) |
| 10 | 0.95 | (0.32 to 2.80) |
| Community* |  |  |
| 1 | Reference |  |
| 2 | 0.55 | (0.14 to 2.09) |
| **3** | **0.16** | **(0.03 to 0.92)** |
| 4 | 0.68 | (0.27 to 1.70) |
| 5 | 0.97 | (0.41 to 2.28) |
| 6 | 0.99 | (0.37 to 2.66) |
| 7 | 1.42 | (0.56 to 3.58) |
| 8 | 1.07 | (0.48 to 2.40) |
| 9 | 1.53 | (0.63 to 3.67) |
| 10 | 1.19 | (0.51 to 2.80) |
| 11 | 0.69 | (0.29 to 1.66) |

* No evidence for a difference between visits/communities using post-hoc Wald test. Coefficients in **bold** where 95% CI do not include unity.

**Supplementary Table S4 - Urinalysis findings at study visits stratified by Hidden Markov Model estimates of kidney status.**

|  | Sex | | | | | | | | | | | |
| --- | --- | --- | --- | --- | --- | --- | --- | --- | --- | --- | --- | --- |
|  | Male | | | | | | Female | | | | | |
|  |  | | | | | |  | | | | | |
|  | Remains healthy | | departure from ~eGFR_(healthy)_ | | Remains unhealthy | | Remains healthy | | departure from ~eGFR_(healthy)_ | | Remains unhealthy | |
| **Urine glucose** |  |  |  |  |  |  |  |  |  |  |  |  |
| Negative | 699 | 99.6% | 18 | 100.0% | 290 | 99.3% | 817 | 99.3% | 6 | 100.0% | 31 | 96.9% |
| 100 mg‎/dL | 1 | 0.1% | 0 | 0.0% | 1 | 0.3% | 1 | 0.1% | 0 | 0.0% | 0 | 0.0% |
| 250 mg‎/dL | 0 | 0.0% | 0 | 0.0% | 1 | 0.3% | 2 | 0.2% | 0 | 0.0% | 0 | 0.0% |
| 500 mg‎/dL | 1 | 0.1% | 0 | 0.0% | 0 | 0.0% | 1 | 0.1% | 0 | 0.0% | 1 | 3.1% |
| 1000 mg‎/dL | 0 | 0.0% | 0 | 0.0% | 0 | 0.0% | 1 | 0.1% | 0 | 0.0% | 0 | 0.0% |
| >2000 mg‎/dL | 1 | 0.1% | 0 | 0.0% | 0 | 0.0% | 1 | 0.1% | 0 | 0.0% | 0 | 0.0% |
| **Urine specific gravity** |  |  |  |  |  |  |  |  |  |  |  |  |
| 1000 | 6 | 0.9% | 0 | 0.0% | 2 | 0.7% | 6 | 0.7% | 0 | 0.0% | 1 | 3.1% |
| 1005 | 11 | 1.6% | 1 | 5.6% | 3 | 1.0% | 10 | 1.2% | 0 | 0.0% | 0 | 0.0% |
| 1010 | 73 | 10.4% | 0 | 0.0% | 46 | 15.8% | 62 | 7.5% | 0 | 0.0% | 4 | 12.5% |
| 1015 | 164 | 23.4% | 7 | 38.9% | 79 | 27.1% | 195 | 23.7% | 1 | 16.7% | 4 | 12.5% |
| 1020 | 197 | 28.1% | 2 | 11.1% | 79 | 27.1% | 241 | 29.3% | 2 | 33.3% | 8 | 25.0% |
| 1025 | 154 | 21.9% | 7 | 38.9% | 56 | 19.2% | 200 | 24.3% | 1 | 16.7% | 10 | 31.2% |
| 1030 | 97 | 13.8% | 1 | 5.6% | 27 | 9.2% | 109 | 13.2% | 2 | 33.3% | 5 | 15.6% |
| **Urine blood** |  |  |  |  |  |  |  |  |  |  |  |  |
| Negative | 651 | 92.7% | 15 | 83.3% | 266 | 91.1% | 594 | 72.2% | 5 | 83.3% | 22 | 68.8% |
| Non-haemolysed trace | 20 | 2.8% | 0 | 0.0% | 8 | 2.7% | 36 | 4.4% | 0 | 0.0% | 0 | 0.0% |
| Non-haemolysed moderate | 3 | 0.4% | 0 | 0.0% | 3 | 1.0% | 3 | 0.4% | 0 | 0.0% | 1 | 3.1% |
| Haemolysed trace | 16 | 2.3% | 1 | 5.6% | 8 | 2.7% | 49 | 6.0% | 0 | 0.0% | 2 | 6.2% |
| Small (+) | 7 | 1.0% | 2 | 11.1% | 5 | 1.7% | 47 | 5.7% | 0 | 0.0% | 3 | 9.4% |
| Moderate (++) | 3 | 0.4% | 0 | 0.0% | 2 | 0.7% | 39 | 4.7% | 0 | 0.0% | 2 | 6.2% |
| Large (+++) | 2 | 0.3% | 0 | 0.0% | 0 | 0.0% | 55 | 6.7% | 1 | 16.7% | 2 | 6.2% |
| **Urine pH** |  |  |  |  |  |  |  |  |  |  |  |  |
| 5.0 | 241 | 34.3% | 7 | 38.9% | 122 | 41.8% | 305 | 37.1% | 2 | 33.3% | 9 | 28.1% |
| 6.0 | 382 | 54.4% | 8 | 44.4% | 130 | 44.5% | 425 | 51.6% | 2 | 33.3% | 21 | 65.6% |
| 6.5 | 65 | 9.3% | 2 | 11.1% | 37 | 12.7% | 72 | 8.7% | 1 | 16.7% | 2 | 6.2% |
| 7.0 | 8 | 1.1% | 0 | 0.0% | 2 | 0.7% | 10 | 1.2% | 1 | 16.7% | 0 | 0.0% |
| 7.5 | 1 | 0.1% | 1 | 5.6% | 1 | 0.3% | 5 | 0.6% | 0 | 0.0% | 0 | 0.0% |
| 8.0 | 5 | 0.7% | 0 | 0.0% | 0 | 0.0% | 4 | 0.5% | 0 | 0.0% | 0 | 0.0% |
| 8.5 | 0 | 0.0% | 0 | 0.0% | 0 | 0.0% | 2 | 0.2% | 0 | 0.0% | 0 | 0.0% |
| **Urine protein** |  |  |  |  |  |  |  |  |  |  |  |  |
| Negative | 691 | 98.4% | 16 | 88.9% | 269 | 92.1% | 809 | 98.3% | 6 | 100.0% | 28 | 87.5% |
| Trace | 7 | 1.0% | 0 | 0.0% | 7 | 2.4% | 8 | 1.0% | 0 | 0.0% | 1 | 3.1% |
| 30 mg‎/dL (+) | 3 | 0.4% | 1 | 5.6% | 9 | 3.1% | 5 | 0.6% | 0 | 0.0% | 1 | 3.1% |
| 100 mg‎/dL (++) | 0 | 0.0% | 0 | 0.0% | 3 | 1.0% | 0 | 0.0% | 0 | 0.0% | 1 | 3.1% |
| 300 mg‎/dL (+++) | 1 | 0.1% | 1 | 5.6% | 4 | 1.4% | 1 | 0.1% | 0 | 0.0% | 1 | 3.1% |
| **Urine nitrite** |  |  |  |  |  |  |  |  |  |  |  |  |
| Negative | 700 | 99.7% | 18 | 100.0% | 291 | 99.7% | 808 | 98.2% | 6 | 100.0% | 31 | 96.9% |
| Positive | 2 | 0.3% | 0 | 0.0% | 1 | 0.3% | 15 | 1.8% | 0 | 0.0% | 1 | 3.1% |
| **Urine leucocytes** |  |  |  |  |  |  |  |  |  |  |  |  |
| Negative | 693 | 98.7% | 18 | 100.0% | 283 | 96.9% | 644 | 78.3% | 6 | 100.0% | 24 | 75.0% |
| Trace | 7 | 1.0% | 0 | 0.0% | 4 | 1.4% | 33 | 4.0% | 0 | 0.0% | 2 | 6.2% |
| Small (+) | 1 | 0.1% | 0 | 0.0% | 3 | 1.0% | 79 | 9.6% | 0 | 0.0% | 5 | 15.6% |
| Moderate (++) | 1 | 0.1% | 0 | 0.0% | 1 | 0.3% | 56 | 6.8% | 0 | 0.0% | 0 | 0.0% |
| Large (+++) | 0 | 0.0% | 0 | 0.0% | 1 | 0.3% | 11 | 1.3% | 0 | 0.0% | 1 | 3.1% |

n, column percent. Grouping based on joint probability >0.5. Individuals may be represented more than once in Healthy and Unhealthy columns at different visits (or not represented in any column if not meeting any of the column criteria). Urinalysis results only available from visit 7.

**Supplementary Table S5 –Exploratory multivariable models of associations with departure from the healthy estimated glomerular filtration rate distribution.**

|  | Model 1,  Odds Ratio  (95%CI) | Model 2,  Odds Ratio  (95%CI) | Model 3  Odds Ratio  (95%CI) | Model 4,  Odds Ratio  (95%CI) | Model 5,  Odds Ratio  (95%CI) | Model 6  Odds Ratio  (95%CI) | Model 7,  Odds Ratio  (95%CI) |
| --- | --- | --- | --- | --- | --- | --- | --- |
| **Cramps, more frequent than 'never or almost never'** | **1.88** | **2.14** | **2.19** | **2.21** | **2.01** | **2.03** | **2.21** |
|  | **(1.12 to 3.15)** | **(1.29 to 3.57)** | **(1.31 to 3.64)** | **(1.33 to 3.69)** | **(1.19 to 3.40)** | **(1.20 to 3.44)** | **(1.33 to 3.70)** |
| Nausea, more frequent than 'never or almost never' | 1.30 |  |  |  |  |  |  |
|  | (0.65 to 2.60) |  |  |  |  |  |  |
| Vomiting, more frequent than 'never or almost never' | 1.57 |  |  |  |  |  |  |
|  | (0.68 to 3.61) |  |  |  |  |  |  |
| Reports of unintentional weight loss | 1.28 |  |  |  |  |  |  |
|  | (0.84 to 1.95) |  |  |  |  |  |  |
| **Weight loss >2.5kg, measured since last visit** | **1.81** | **1.91** | **1.87** | **1.88** | **1.88** | **1.91** | **1.86** |
|  | **(1.10 to 2.99)** | **(1.15 to 3.17)** | **(1.12 to 3.11)** | **(1.12 to 3.14)** | **(1.13 to 3.12)** | **(1.14 to 3.21)** | **(1.11 to 3.11)** |
| NSAIDs, regularly or more frequently since last visit |  | 1.94 |  |  |  |  |  |
|  |  | (0.94 to 4.01) |  |  |  |  |  |
| **Sugarcane work since last visit** |  |  | **1.70** |  | **1.67** | 1.58 | 1.58 |
|  |  |  | **(1.05 to 2.75)** |  | **(1.03 to 2.70)** | (0.96 to 2.59) | (0.97 to 2.57) |
| Symptoms of excess occupational sun exposure, since last visit |  |  |  | 1.41 | 1.37 | 1.34 |  |
|  |  |  |  | (0.89 to 2.24) | (0.86 to 2.18) | (0.83 to 2.17) |  |
| Work outdoors regularly or more frequently, since last visit |  |  |  |  |  | 1.35 |  |
|  |  |  |  |  |  | (0.75 to 2.45) |  |
| Shade available at work, since last visit |  |  |  |  |  | 0.46 |  |
|  |  |  |  |  |  | (0.13 to 1.64) |  |
| Work breaks in the shade, since last visit |  |  |  |  |  | 0.90 |  |
|  |  |  |  |  |  | (0.31 to 2.59) |  |
| Work in a very hot environment regularly or more frequently |  |  |  |  |  | 0.78 |  |
|  |  |  |  |  |  | (0.47 to 1.27) |  |
| Daily water intake (litres), since last visit |  |  |  |  |  |  | 1.06 |
|  |  |  |  |  |  |  | (0.96 to 1.17) |

NSAID, non-steroidal anti-inflammatory drug. Adjusted for age, sex, study visit and follow-up duration in addition to all variables where coefficients are presented. The models were developed to explore the following: Whether cramps, nausea, vomiting and weight loss were independently associated with the outcome (Model 1); Whether cramps and weight loss were surrogates for NSAID use (Model 2);Whether cramps and weight loss were surrogates for recent work in sugarcane (Model 3); Whether cramps, weight loss and sugarcane work were surrogates for occupational sun exposure or heat stress more generally (Model 4 to 6) or dehydration, estimated by self-reported water intake (Model 7). Coefficients in **bold** where 95% CI do not include unity.

**Supplementary Table S6 – Exploration of proportion reporting cramps, occupational sun exposure and measured weight loss by season and sex**

|  | Male | | | | Female | | | |
| --- | --- | --- | --- | --- | --- | --- | --- | --- |
|  | Pre-harvest | | Post-harvest | | Pre-harvest | | Post-harvest | |
| Cramps, more frequent than 'never or almost never'^†^ | 56/712, | 7.9% | 53/476, | 11.4% | 18/238, | 7.3% | 20/158, | 12.7% |
| Symptoms of excess occupational sun exposure^‡^ | 95/712, | 13.3% | 121/476, | 26.0% | 9/246, | 3.7% | 28/158, | 17.7% |
| Weight loss >2.5kg, measured since last visit*^¶^ | 76/469, | 16.2% | 43/444, | 9.7% | 30/162, | 18.5% | 21/156, | 13.5% |

Cohort 1, first 5 (bi-annual) visits only. Visits 2 and 4 were post-harvest. *No data at Visit 1; ^†^*P=0.008* for a seasonal difference *P=NS* for a sex difference; ^‡^*P<0.001* for a seasonal difference and a sex difference: ^¶^*P<0.002* for a seasonal difference and *P=NS* for a sex difference. P-values for Chi=squared.

**Supplementary Figure S1 – Spaghetti plots of individual eGFR trajectories stratified by healthy-unhealthy status and sex in Cohort 1(i) and 2(ii).** Individual eGFR trajectories grouped by estimates HMM state (posterior probability >0.5) at baseline and final follow-up. Males: left, females: right. (ia) and (iia) healthy throughout; (ib) and (iib) healthy at baseline, unhealthy at last HMM visit; (ic) and (iic) unhealthy throughout, (id) participants who revert from unhealthy to healthy at any point during follow-up. Red crosses show timepoint of probable departure from ~eGFR_(healthy)_ (joint probability >0.5; b and d only).

**
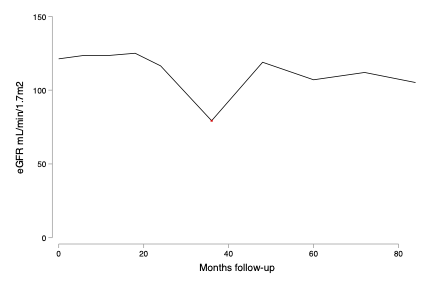

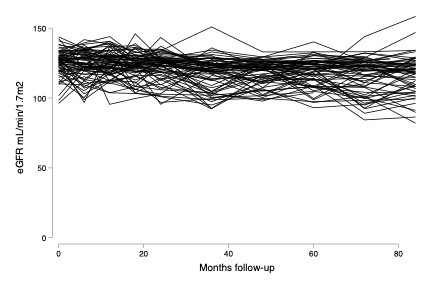

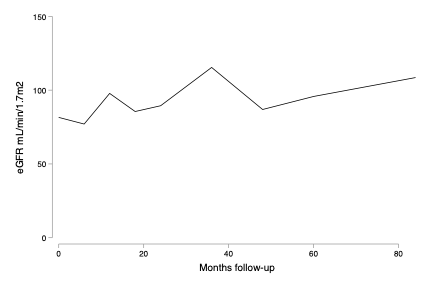

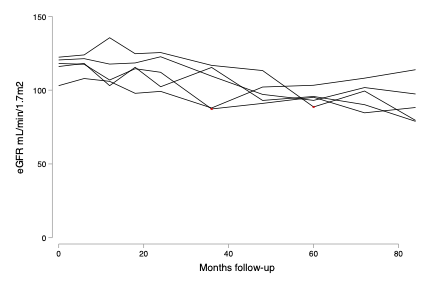

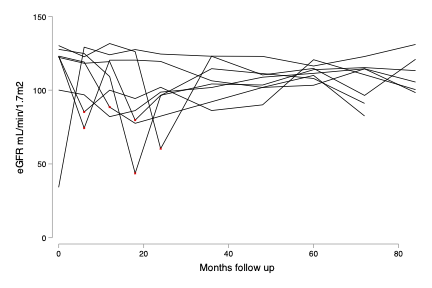

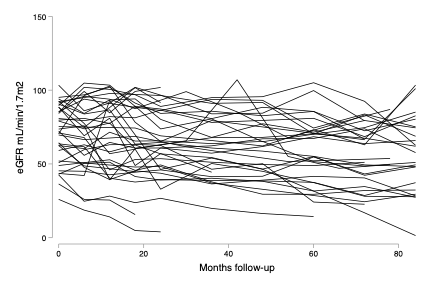

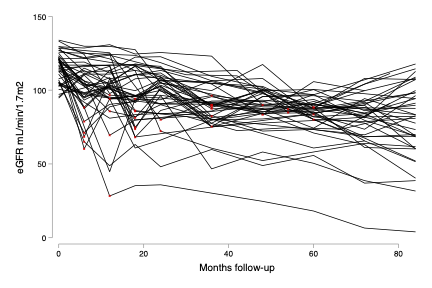

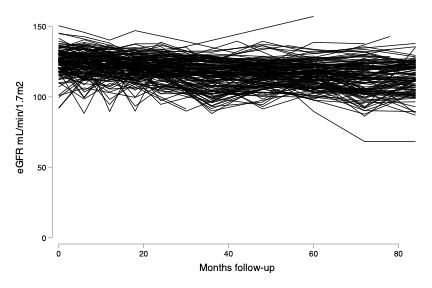

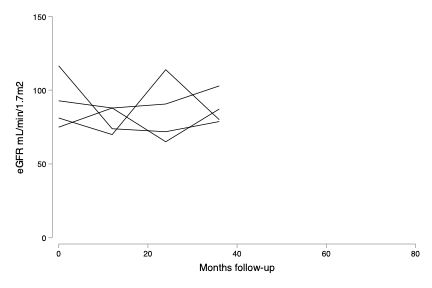

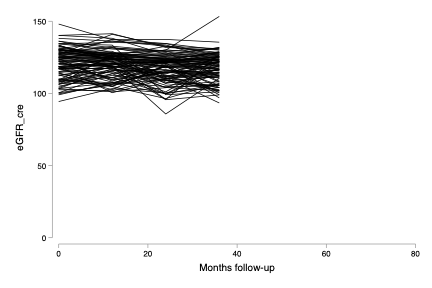

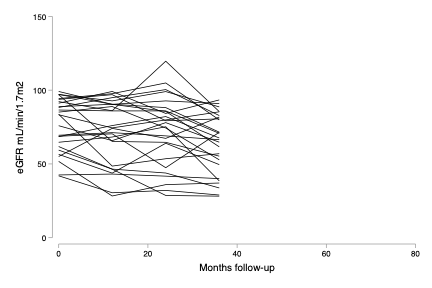

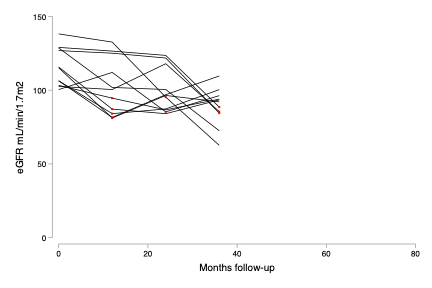

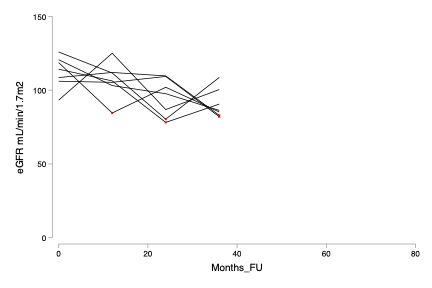

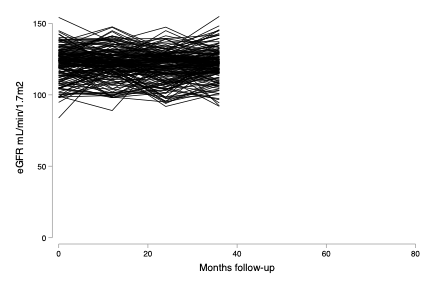
**

(id)

(ic)

(ib)

(ia)

(iic)

(iib)

(iia)

**Supplementary Figure S2 - Distribution of estimated posterior probabilities of unhealthy state from Hidden Markov Model by study visit in Cohort 1 and 2.** The figure shows the distribution of the estimated posterior probabilities of being in the unhealthy kidney state for each individual visit. For example, an individual with an eGFR of 120 at Visit 1 will have a near 0 estimated posterior probability of being in the unhealthy state at Visit 1. An individual with an eGFR value of around 90 will have a higher posterior probability of being unhealthy at that point in time. The posterior probability is the probability that an individual is in a state at the visit based on the fit model and the observed eGFR at that visit i.e., P(Unhealthy at a visit | observed eGFR).

**Supplementary Figure S3 – Distribution of estimated probability of sustaining departure from ~eGFR_(healthy)_ derived from the Hidden Markov Model by study visit in Cohort 1 and 2.** The figure shows the distribution of the estimated joint probabilities of transitioning from a healthy state to an unhealthy for each individual visit. In contrast to Figure 1, this joint probability takes into account eGFR observations at two subsequent visits. It includes the probability of having been in a healthy state and the probability of going to an unhealthy state conditional on being in the healthy state at the previous visit that is the joint probability conditional on the eGFR observations i.e., P(Unhealthy now and Healthy at previous visit) = P(Unhealthy now | Healthy at previous visit)P(Healthy at previous visit )

**Supplementary Figure S4 – QQ Plots showing random effects parameters (top and middle) and residuals (bottom) from the linear mixed model.** Model specific with eGFR measures nested within individuals and a random intercept and slope for time. Adjusted for age and sex. Random effects parameters are non-normally distributed demonstrating poor model fit.
